# Reversible Pancytopenia Due to Severe Iron Deficiency Anemia in a 12-Year-Old Girl

**DOI:** 10.7759/cureus.62175

**Published:** 2024-06-11

**Authors:** Tahlil Al Amri, Issa Al Mamari, Aza S AlSawafi, Muntadhar Al Moosawi, Abeer Al-Battashi

**Affiliations:** 1 Child Health, Oman Medical Specialty Board, Muscat, OMN; 2 Medicine, Sultan Qaboos University Hospital, Muscat, OMN; 3 Pediatric Medicine, The Royal Hospital, Muscat, OMN; 4 Hematopathology, The Royal Hospital, Muscat, OMN; 5 Pediatric Hematology and Oncology, The Royal Hospital, Muscat, OMN

**Keywords:** iron deficiency, cytopenia, pediatrics, anemia, pancytopenia

## Abstract

Iron deficiency anemia remains a global health challenge among young children starting from birth. Pancytopenia is a rare presentation of iron deficiency in children, being highly reversible with a simple treatment strategy including diet modification and iron supplementation.

A 12-year-old Omani girl presented with a four-week history of fatigue, dizziness, and palpitations. Investigations revealed hemoglobin of 3.3 g/dL (11-14.5 g/dL), platelet of 47×10^9^/L (150-450×10^9^/L), white cell count of 3.9×10^9^/L (2.4-9.5×10^9^/L), and absolute neutrophil count of 0.5×10^9^/L (1-4.8×10^9^/L). Her red blood cell distribution width was high (34.1%; 11.5-16.5%), and her reticulocytes were normal (2.1%; 0.2-2%) with a mild elevation in the immature reticulocytes. Moreover, the ferritin level was profoundly low with a result of 1 ug/L only. Her peripheral blood smear showed pancytopenia in a background of microcytic and hypochromic red cells. After appropriate supplementation with oral ferrous sulfate in combination with an iron-rich diet, she showed complete recovery after six months from the initiation of management.

This case report highlights that iron deficiency can be a cause of severe pancytopenia in children.

## Introduction

Pancytopenia can arise from a variety of etiologies, including bone marrow suppression, systemic infections, malignancies, and nutritional deficiencies. Iron deficiency anemia (IDA), while a leading cause of anemia globally, infrequently leads to pancytopenia. The pathophysiology of IDA involves inadequate dietary intake, malabsorption, or chronic hemorrhage, critically impairing hemoglobin synthesis and cellular oxygen transport [1,2].

The manifestation of pancytopenia within the framework of IDA remains exceedingly rare and underexplored. The literature review indicates that only a few cases have been documented, highlighting the rarity of this clinical presentation. Due to its infrequency, comprehensive epidemiological data on IDA-associated pancytopenia is scarce, with most knowledge derived from isolated case reports and limited case series. These reports suggest a wide variability in clinical presentation, ranging from asymptomatic laboratory findings to severe manifestations, including symptoms of anemia, infection susceptibility, and hemorrhagic tendencies [3,4].

In pathophysiological terms, the link between iron deficiency and pancytopenia is not fully elucidated but is hypothesized to involve iron's pivotal role in DNA synthesis and cellular proliferation within the bone marrow. The prognosis for patients with IDA-induced pancytopenia is generally favorable, with iron supplementation leading to the resolution of hematologic abnormalities. This reversibility underscores the critical importance of early detection and treatment initiation [5].

This case report describes a rare presentation of a common phenomenon in pediatric patients, delineating the fascinating presentation and good outcome. Verbal informed consent was obtained from the guardian of this patient.

This article was presented as an abstract at the Kuwait International Multidisciplinary Pediatric Conference, on February 1, 2024.

## Case presentation

History and physical examination

A 12-year-old Omani girl presented to the emergency department in 2023, complaining of progressive pallor, fatigue, dizziness, and intermittent palpitations that had been going on for a month. She did not experience any weight loss or fever and had not recently traveled outside her country or taken any medication or herbal supplements. She had menarche at the age of 11 years and had regular periods without any abnormal bleeding. Her family history was unremarkable except for glucose-6-phosphate dehydrogenase deficiency. The patient has a limited diet and mainly consumes small amounts of meat and vegetables.

The patient's vital signs were normal during the clinical examination. She was remarkably pale, and there were no signs of jaundice. The patient did not exhibit any physical abnormalities, like palpable lymph nodes, liver, or spleen, or any developmental delays for her age. Additionally, there were no petechiae or bruises, rashes, or oral ulcerations.

Investigations and management

The initial investigations (Table 1) showed microcytic hypochromic anemia with hemoglobin of 3.3 g/dL, thrombocytopenia of 47×10^9^/L, leukopenia of 3.9×10^9^/L, and absolute neutrophil count of 0.5×10^9^/L. The red blood cell distribution width was increased, 34.1%, and there was no reticulocytosis. The urea, creatinine, electrolytes, bilirubin (total and conjugated), and liver enzymes were all within biological ranges. Stool microscopy was negative for any parasitic infestation. Uric acid and lactate dehydrogenase results were normal, suggesting no underlying abnormal cellular turnover. In addition, vitamin B12 and folate levels were normal. The serum ferritin was found to be profoundly low at 1 ug/L.

**Table 1:** Laboratory parameters indicative of pancytopenia and iron deficiency upon presentation and after initiation of therapy. MCV: mean corpuscular volume; MCH: mean corpuscular hemoglobin; MCHC: mean corpuscular hemoglobin concentration; RDW: red blood cell distribution width

Laboratory parameter	At presentation	1 week	2 weeks	1 month	Unit	Range
Hemoglobin	3.3	7.4	7.4	12.2	g/dL	11.5-15.5
Hematocrit	12.9	24.9	24.1	40.5	%	35-45
Red blood cells	2.8	4.36	4.33	5.97	×10^12^/L	2.9-5.3
MCV	46.0	57.2	55.7	67.9	fL	73-95
MCH	11.9	17.0	17.1	20.5	pg	24-33
MCHC	25.8	29.8	30.7	30.2	g/dL	31-35
RDW	34.1	34.0	36.0	20.6	%	11.5-16.5
Platelet count	47	800	1013	300	×10^9^/L	150-450
White cell count	3.9	6.1	4.5	4.9	×10^9^/L	4.5-14.5
Neutrophils	0.5	4.2	1.5	1.7	×10^9^/L	1.4-9
Lymphocytes	3.0	0.9	2.1	2.6	×10^9^/L	1.9-9.8
Monocytes	0.2	0.9	0.5	0.4	×10^9^/L	0.1-1.3
Reticulocyte	2.1	4		2.0	%	0.2-2.0
Immature reticulocyte	0.43	0.42		0.3	%	0.1-0.3
Ferritin	1	4			ug/L	10-291

**Figure 1:**
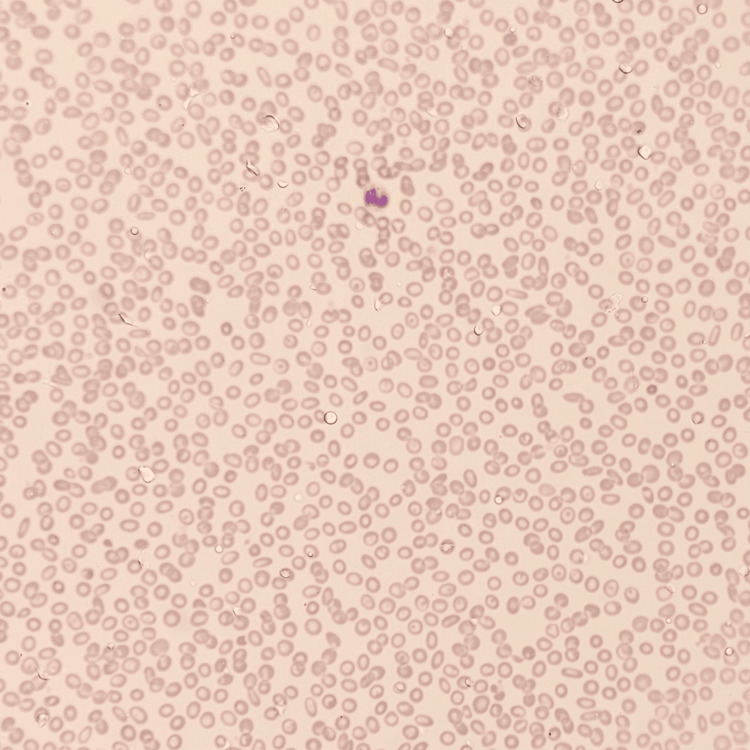
Blood film findings of severe hypochromia and microcytosis, thrombocytopenia, and leukopenia.

The blood film (Figure 1, original magnification ×40; Wright-Giemsa stain) showed an overall picture of pancytopenia with profound microcytosis and hypochromia with poikilocytosis including elliptocytes, pencil cells, tear drop cells, target cells, and rare spherocytes. In addition, the high-performance liquid chromatography (HPLC) identified no abnormal hemoglobin variant, and the direct Coombs test (DCT) was negative.

Additional investigations for pancytopenia were found to be normal including erythrocyte sedimentation rate and anti-nuclear antibodies. The celiac disease work-up (anti-tissue transglutaminase antibody in serum) was negative of less than 2 U/mL. In addition, parvovirus, cytomegalovirus, and Epstein-Barr virus serologies were all negative. The chest X-ray was normal, and the abdominal ultrasound showed mild hepatomegaly with no focal lesions and normal rest of structures.

After admission, the patient received a packed red blood cell transfusion (15 ml/kg), after which her hemoglobin improved to 6.9 g/dL. The patient was then started on an oral ferrous sulfate supplement of 6 mg/kg/day for three months. She was also counseled by a dietitian to improve her diet and increase her iron-rich food intake. She remained well and active throughout her admission for a period of three days.

One week into her supplementation and diet modification, she showed improvement in symptoms with normal clinical examination. Serial blood investigations (Table 1) showed an improvement in hemoglobin to 7.4 g/dL, thrombocytosis of 800×10^9^/L, and normal leukocyte count (and absolute neutrophil count). Reticulocyte recovery to 4% accompanied the overall improvement.

A repeat blood film after 12 weeks showed a dimorphic blood picture with normocytic and normochromic red cells as well as a population of microcytic and hypochromic red cells. There was occasional polychromasia. The blood film also showed marked thrombocytosis, many large platelets, and an occasional giant platelet. This might be an indication of a mixed blood film due to bone marrow recovery in combination with previous exposure to blood transfusion.

A month later, she came to the outpatient department looking well. The hemoglobin normalized to 12.2 g/dL, with a total resolution of other cytopenias. She went home to complete three months of oral ferrous sulfate supplements (300 mg per day which is equivalent to 60 mg per day of elemental iron). She has been followed up for three years with no clinical concerns and no abnormal laboratory results.

## Discussion

The most common cause of anemia in developing nations is iron deficiency [2]. It is commonly described due to chronic blood loss, for example, due to hookworm infestation or nutritional compromise. Pancytopenia is a condition that is categorically comprised of abnormally low hemoglobin levels for age and sex, thrombocytopenia, and leukopenia.

Pancytopenia in children is mainly secondary and commonly caused by infection or inflammation. Other causes include bone marrow infiltration, like leukemia or metastasis. Inherited and idiopathic aplastic anemia is rare but a serious etiology of such presentation. In some parts of the developing world, nutritional deficiencies were found to be a common cause of pancytopenia, with megaloblastic anemia reported as the most common [6,7].

There are very few cases of similar presentations that were reported in the literature [8,9]. Meena et al. have reported six adult patients with pancytopenia due to IDA, with a hemoglobin range of 3.1-6.8 g/dL, platelet range of 30-99×10^9^/L, and white cell count of 2-3.7×10^9^/L [9]. All patients recovered after four weeks of parenteral iron supplementation with a good outcome.

It is proven that bone marrow iron metabolism plays a major role in hematopoiesis, whether undergoing malignant or benign transformation through different regulatory pathways starting from the enterocytes of the duodenum, moving to the liver, the kidneys, and the bone marrow microenvironment [10]. For instance, hepcidin, which is a peptide synthesized within the liver, is considered to be one of the most important master regulators that responds to fluctuations of iron levels outside the normal homeostatic range [10]. The exact mechanism of thrombocytopenia and leukopenia in IDA is not very well established [4,5,11]; however, it has been demonstrated that iron deficiency can affect the megakaryocytic erythroid progenitors within the bone marrow directly by affecting the intracellular signaling pathways responsible for differentiation and cellular division [10]. Although mild IDA can lead to thrombocytosis, profound iron loss can lead to the contrary, thrombocytopenia, like in this patient. In addition, isolated thrombocytopenia in the context of IDA is a very rare presentation [12]. A general rule is that all patients with any type of cytopenia must undergo an iron level testing to rule out this common etiology-associated rare presentation.

## Conclusions

This patient revealed the rare faces of IDA in children. Physicians must be very careful approaching childhood pancytopenia and must rule out ominous causes whenever the clinical condition warrants. A careful clinical assessment followed by appropriate iron supplementation can lead to favorable recovery, avoiding invasive interventions, including bone marrow aspiration and gastrointestinal endoscopies.
